# Identification of Optimal Fermentation Temperature for Dry-Fermented Sausage Using Strains Isolated from Korean Fermented Foods

**DOI:** 10.3390/foods12010137

**Published:** 2022-12-27

**Authors:** Chang-Hwan Jeong, Sol-Hee Lee, Yohan Yoon, Hyung-Youn Choi, Hack-Youn Kim

**Affiliations:** 1 Department of Animal Resources Science, Kongju National University, Yesan-Gun 32439, Republic of Korea; 2 Department of Food and Nutrient, Sookmyung Women’s University, Seoul 04310, Republic of Korea; 3 Food Standard Research Center, Food Industry Research Division, Korea Food Research Institute, Wanju-Gun 55365, Republic of Korea

**Keywords:** dry-fermented sausage, fermentation temperature, flavor, microbial population, Korean fermented food

## Abstract

This study aims at identifying the optimal fermentation temperature for dry-fermented sausage using strains isolated from *Kimchi* (GK1, *Pediococcus pentosaceus*-GK1; NK3, *P. pentosaceus*-NK3), *Doenjang* (D1, *Debaryomyces hansenii*-D1), and commercial fermented sausage (S6, spontaneously generated *Penicillium nalgiovense*-S6). The microbial population, pH, moisture content, color, thiobarbituric acid reactive substance (TBARS), volatile basic nitrogen (VBN), and electronic nose (E-nose) were analyzed to identify the optimal fermentation temperature. The dry-fermented sausages were inoculated with three types of starter cultures [CS (commercial starter culture), GD (GK1 + D1 + S6), and ND (NK3 + D1 + S6)]. The fermentation was performed for 3 days at 20 °C and 25 °C, and dried for 28 days. The *Lactobacillus* spp. plate count and TBARS showed significantly higher values in the 25 °C group than in the 20 °C group (*p* < 0.05). The *Staphylococcus* spp. plate count of GD and ND were significantly higher than CS group at all temperatures. On day 31, the moisture content and VBN values of all groups were less than 35 % and 20 mg%, respectively. According to E-nose, the highest amount of acetoin was detected at the GD group fermented at 25 °C. Thus, the optimal fermentation temperature is expected at 25 °C after using GD in the manufacturing of dry-fermented sausages.

## 1. Introduction

Fermented sausage is a representative fermented meat product manufactured by mixing pork, fat, glucose, spices, and a starter culture consisting of lactic acid bacteria (LAB), yeast and mold, followed by fermentation and dry aging [1]. In fermented sausages, LAB produces lactic acid from added sugar and lowers the pH, inhibiting the growth of pathogenic and spoilage microorganisms while decomposing fat and protein [2,3]. The endogeneous enzymes and microbial enzymes are primarily responsible for the proteolysis and lipolysis. These enzymes breakdown proteins and lipids into free amino acids (FAAs) and free fatty acids (FFAs) [4]. FAAs not only form the taste of fermented sausages but can also be metabolized into compounds that contribute to the unique flavor of fermentation, such as aldehydes, alcohols, and alkanes [5]. In addition, moderate oxidation of FFAs derived from lipolysis plays a significant role in flavor development [6].

The microorganisms in *Kimchi* and *Doenjang*, which are Korean fermented foods, break down proteins to produce large amounts of glutamic acid, alanine, and glycine [7,8]. These free amino acids can improve the taste and flavor of fermented sausages, and the degree of expression varies according to the composition of various microorganisms. In *Kimchi* and *Doenjang*, various microbial groups such as *Lactilactobacillus sakei*, *Pediococcus pentocaceus*, *Weissella cibaria*, *Debaryomyces hansenii*, and *Saccharomyces cerevisiae* are present [9,10]. Yeong et al. [11] and Jeong et al. [12] determined that *Weissella koreensis* spp. and *Staphylococcus* spp. isolated from *Kimchi* and *Doenjang*, respectively, had microbiological stability. Therefore, the strains isolated from Korean fermented foods have high potential as a starter culture of fermented sausage.

Various studies have shown the trend of using a combination of LAB, coagulase-negative staphylococci (CNS), yeast, and mold in the manufacture of fermented sausages. Chen et al. [13] reported that the fermented sausage inoculated with *Lactiplantibacillus plantarum* and *Staphylococcus xylosus* showed effects of inhibiting fat rancidity and enhancing flavor compared to the fermented sausage inoculated with *L. plantarum*. Cebrián et al. [14] reported that the mixed inoculation of *D. hansenii* and *S. xylosus* in fermented sausages inoculated already with *Penicillium nordicum* had a superior ochratoxin A, an inhibitory effect, compared to that inoculated with *S. xylosus* alone. Therefore, mixed inoculation with starter culture increases the microbiological safety of fermented sausages. 

The physicochemical and sensory characteristics of fermented sausages are mainly influenced by the growing bacterial diversity and fungal communities [15,16]. Fermented sausages exhibit different microbial compositions depending on fermentation conditions such as temperature and relative humidity, and are particularly sensitive to temperature due to the different optimal growth conditions for each microorganism [17,18]. Therefore, identifying the temperature at which all strains can grow properly is important when mixing and using the starter culture of fermented sausage. Therefore, this study aims to identify the optimal fermentation temperature after addition of the strain isolated from Korean fermented foods to fermented sausages.

## 2. Materials and Methods

### 2.1. Strain Preparation

In this study, *P. pentosaceus* SMFM2016-GK1 (GK1) and *P. pentosaceus* SMFM2016-NK3 (NK3) isolated from *Kimchi*; *D. hansenii* SMFM2021 D1 (D1) isolated from doenjang; and *Penicillium nalgiovense* SMFM2021 S6 (S6) isolated from fermented sausages were used. The strains identified via 16S rRNA (lactic acid bacteria) sequencing and 26s rRNA sequencing (yeast and mold) were supplied by Sookmyung Women’s University (Seoul, Korea). The lactic acid bacteria were cultured for 1 day at 37 °C using de Man–Rogosa–Sharp (MRS) broth. The yeast was cultured for 2 days at 25 °C using yeast malt (YM) broth. Each broth was freeze-dried after removing the supernatant and stored at −90 °C. The mold was incubated for 3 days at 25 °C after inoculating the strain on potato dextrose agar (PDA). Then, spraying 10 mL of sterile saline solution on PDA incubated mold, it was mixed and used. For the control group, the *Lactobacillus* powder (21 mixed lactic acid bacteria, Biotopia, ChunCheon, Korea) in which 5 types of *Lactobacillus spp.*, 3 types of *Lacticaseibacillus* spp., 2 types of *Limosilactobacillus* spp., 1 type of *Lactiplantibacillus* spp., 1 type of *Levilactobacillus* spp., 1 type of *Ligilactobacillus* spp., 5 types of *Bifidobacterium* spp., 2 types of *Leuconostoc* spp., and 1 type of *Streptococcus* spp. are mixed, and *P. nalgiovense* Sarterkulturen Edelschimmel is supplied by the National Institute of Animal Science (Wanju, Korea).

### 2.2. Sausage Manufacturing and Sampling

For raw meat, pork hind legs (Landrace × Yorkshire × Duroc, each weighing approximately 110 kg) 24 h after slaughter were purchased from I-homemeat (Seoul, Korea). The pork hind legs were used to make sausages after removing excess fat and connective tissue. The pork hind leg and back fat were pulverized to a size of 3 mm. Each sausage dough is prepared by mixing pork hind legs (4250 g), pork back fat (750 g) and the following additives (g/kg meat): salt (100), black pepper (15), ascorbic acid (1.5), glucose (40), garlic (25), bird’s eye chili (5), red wine (100), and starter culture (3). Sausage dough was filled into a 40 mm diameter fibrous casing weighing approximately 250–300 g, and after rinsing, a hole was made using a sausage pricker. After suspension, the sausage was sprayed back and forth twice with a sprayer containing liquid mold.

Subsequently, they were divided into six batches with different combinations of three types of inoculated starter culture (CS, GD, and ND) at fermentation temperatures (20 °C and 25 °C). The starter cultures used the combination that showed the optimal sensory properties in the previous study [19]: (1) CS20 (Commercial starter culture fermented at 20 °C), (2) CS25 (Commercial starter culture fermented at 25 °C), (3) GD20 (GK1 + D1 + S6 fermented at 20 °C), (4) GD25 (GK1 + D1 + S6 fermented at 25 °C), (5) ND20 (NK3 + D1 + S6 fermented at 20 °C), and (6) ND25 (NK3 + D1 + S6 fermented at 25 °C). Each strain was adjusted to a level of 8 log colony-forming units (CFU)/g meat. Sausages were fermented for three days at temperatures 20 °C and 25 °C and 70% relative humidity (RH), and finally dried at 14 °C and 68% RH for 28 days while the temperature and humidity decreased gradually. In this experiment, sausages were collected and used every 0, 3, 10, 17, 24, and 31 days. The microbial population was analyzed including the casing of the sausage, and pH, moisture content, thiobarbituric acid reactive substances (TBARS), volatile basic nitrogen (VBN), and electronic nose were used after removing the casing.

### 2.3. Microbial Analysis

As for the microbial population, the plate counts of aerobic bacteria (AC), *Lactobacillus* spp. (LABC), *Staphylococcus* spp. (STPC), and of yeast and mold (YMC) were measured. After placing 25 g sample and 50 mL sterile saline solution in a sterile bag, it was homogenized for 1 min. A diluted solution was prepared by mixing 1 mL of the homogenate with 9 mL of sterile saline solution and diluting as necessary. The diluted solution was dispensed to 3M^TM^ Petrifilm (3M, Saint Paul, MN, USA) for AC, MRS agar for LABC, Mannitol salt agar (MSA) for STPC, and Potato Dextrose Agar (PDA) for YMC. The aerobic bacteria, *Lactobacillus* spp., and *Staphylococcus* spp. were incubated at 37 °C for 24 h, and yeast and mold were incubated at 25 °C for 48 h. The number of colonies cultured was then measured and expressed as log CFU/g.

### 2.4. pH

Using ultra turrax (HMZ-20DN, Pooglim Tech, Seongnam, Korea), 3 g of sausage and 12 mL of deionized water were homogenized for 1 min (6451× *g*). The homogenate pH was measured using a pH meter (Model S220, Mettler-Toledo, Greifensee, Switzerland) calibrated with buffer solutions (pH: 4.01, 7.0, and 10.0).

### 2.5. Moisture Content

The moisture content of sausages was measured according to the Association of Official Analytical Chemists (AOAC) guidelines [20]. After placing 1 g of the sample in a drying oven (C-F03; Vision Scientific, Daejeon, Korea) set at 105 °C, it was dried for 18 h. The equation for moisture content calculation was as follows:

$$\mathrm{Moisture}\;\mathrm{content}\;\left(\%\right)=\frac{W_{1}-W_{2}}{W_{1}}\times 100$$

where *W*_1_ and *W*_2_ are weights before and after drying, respectively.

### 2.6. Color

The cross section of the sausage was measured using a colorimeter (CR-10, Minolta, Tokyo, Japan), with an 8 mm measuring diameter and an 8 lx illumination angle (CIE standard illuminant D_65_). The lightness, redness, and yellowness were indicated as CIE L^*^, CIE a^*^, and CIE b^*^, respectively, and a white standard plate (CIE L^*^: +97.83; CIE a^*^: –0.43; CIE b^*^: +1.98) was used as the standard color.

### 2.7. TBARS

The TBARS of the sausage was determined using the method by Jeong et al. [21]. Using a homogenizer (AM-5, Nihonseiki Kaisha, Tokyo, Japan), 10 g of the sample, 200 µL of 0.3% butylated hydroxytoluene (BHT), and 50 mL of deionized water were homogenized for 2 min (5614× *g*). The homogenate was mixed with 2.5 mL of 4 N hydrochloric acid, 47.5 mL of deionized water, a boiling stone, and 1 mL of an antifoaming agent. It was then heated with a heating mantle (MS-E102, Lab Merchant, London, UK) set at 100 °C. After that, the distillate was collected and used as a sample. The sample was mixed with 0.02 M 2-thiobarbituric acid in 90% acetate in a 1:1 ratio, heated in a water bath (JSWB-30T, JSR, kongju, Korea) set at 100 °C for 35 min, and cooled in cold water for 10 min. Afterward, it was measured at 538 nm using a multi-mode microplate reader (Spectra Max ID3, Molecular Devices, San Jose, CA, USA), and the measured value was substituted into the standard curve to convert it to the amount of malondialdehyde, where 1,1,3,3-tetraethoxypropane was used as the reference material.

### 2.8. VBN

The VBN of the sausage was measured by partially modifying the method of Conway and O’Malley [22]. Using a homogenizer (AM-5, Nihonseiki Kaisha) (5614× *g*), 10 g of sample and 30 mL of deionized water were homogenized for 1 min. After filtration with filter paper (Whatman No. 1, Whatman, Maidstone, UK), it was used as a sample. A 100 µL of 0.01 N boric acid and Conway indicator (0.066% methyl red + 0.066% bromocresol green + ethanol) were added to the inner of the Conway dish, and to the outer, 1 mL sample and 1 mL 50% potassium carbonate were added. Then, 1 mL of deionized water was added as a blank sample. After the vaseline-coated Conway dish lid was covered and reacted at 37 °C for 2 h, 0.02 N sulfuric acid was titrated until the solution inside the Conway dish turned from green to red. The equation for VBN content calculation was as follows:

$$\mathrm{VBN}\;\left(\mathrm{mg}\%\right)=\frac{V_{1}\;\left(\mu L\right)-V_{2}\;\left(\mu L\right)}{m\;\left(g\right)}\times 0.14\times a\times b\times 100$$

where *V*_1_ and *V*_2_ are titration volumes of sample solution and blank, respectively; *m* is the weight of sample; *a* is titer value of 0.02 N sulfuric acid; and *b* is the dilution factor.

### 2.9. Electronic Nose (E-Nose)

E-nose was used to analyze the aroma profile of sausage using the Go et al. [23] method. The sample was prepared by putting 5 g of sausage into a vial. An E-nose system (Heracles-II-e-nose, Alpha MOS, Toulouse, France) equipped with two columns (MXT-5/MXT-1701, Restek, Bellefonte, PA, USA) was used for measurement. E-nose testing conditions were as follows: injection speed, 125 µL/s; injection temperature, 200 °C; trap absorption temperature, 80 °C; trap desorption temperature, 250 °C; and acquisition time, 110 s. The measured values were expressed in the form of principal component analysis (PCA) and volatile compound peaks using the alpha software program (Alpha MOS, Toulouse, France).

### 2.10. Statistical Analysis

All data in this study were output by repeating experiments at least 3 times. All data were presented as the mean value and standard errors of the means (SEM), and one-way analysis of variance (ANOVA) using the General Linear Models procedure in the SAS program (version 9.4 for window, SAS Institute, Cary, NC, USA). Significant differences between data were analyzed using Duncan’s multiple range test (*p* < 0.05).

## 3. Results and Discussion

### 3.1. Microbial Population

Table 1 shows the microbial population according to the fermentation temperature of fermented sausages using strains isolated from Korean fermented foods. The aerobic plate count (AC) of all groups increased and then decreased until days 10 to 17. Microorganisms in fermented sausages show a rapid increase during fermentation owing to suitable fermentation temperature and RH [24]. However, the reduction of sugar with the growth of microorganisms and the large amount of carbon dioxide produced during the fermentation process provide an unfavorable environment for aerobic bacteria growth [25]. Therefore, the AC values of all groups tended to decrease after day 17. The AC values of GD, CS, and ND groups were significantly higher at 25 °C than that of 20 °C until days 3, 10, and 31, respectively (*p* < 0.05). Agüero et al. [26] reported that *Lactobacillus helveticus* R0052, *Limosilactobacillus reuteri* DSM17918, and *L. reuteri* DSM17938 did not grow at specific temperatures when commercial probiotic bacteria were cultured at various temperatures. Accordingly, this phenomenon can be observed in the microbial group of ND group compared to the GD and CS groups owing to the different growth conditions based on the temperatures applied. In addition, the LABC values of all groups were significantly higher at 25 °C than that at 20 °C in all periods (*p* < 0.05). For short-term aging fermented sausages, breaking down proteins and fats at a rapid rate is necessary through the rapid growth of lactic acid bacteria [27]. In the GD group, GD25 showed a faster growth rate than that of GD20, and in the ND group, ND25 decreased at a slower rate than that of ND20 after the peak growth point. Wang et al. [28] reported that the optimal growth temperature for lactic acid bacteria varies depending on the species. As the LABC value of the 25 °C group was higher than that of the 20 °C group, the fermentation of the lactic acid bacteria (CS, GK1, and NK3) used in this study at 25 °C was suitable to produce short-term aging fermented sausages. The STPC of the CS group was significantly lower than that of the GD and ND groups in all periods, which may have been caused by the growth of CS. Xiao et al. [29] reported that *Staphylococcus* spp. was sensitive to low-level pH environments. As the CS group showed a higher LABC value than that of GD and ND groups, the pH in the fermented sausage may have been relatively low, leading to the low STPC value. On day 3, the YMC values of CS25 and GD25 were significantly higher than those of CS20 and GD20, and ND25 showed a significantly lower value until the day 10 compared to ND20 (*p* < 0.05). However, as the fermentation and drying periods elapsed, CS25 and GD25 showed lower YMC values compared to that of CS20 and GD20. It is judged that the 25 °C group has a lower YMC value than the 20 °C group because lactic acid bacteria show a superiority over yeast and mold. The ND group, however, did not show a significant difference between the temperatures after the 17th day, and it seemed to have less competition between yeast and mold. Furthermore, yeast and mold had a lower optimal growth temperature than that of lactic acid bacteria, and fermentation at 20 °C provided a suitable environment for yeast and mold to grow.

According to microbial population analysis results, the 25 °C group showed higher levels of AC, LABC, and STPC, except for YMC, than that of 20 °C group. As the rapid growth rate of *Lactobacillus* spp. was required in short-term aged fermented sausages, fermentation at 25 °C seemed appropriate for both GD and ND.

### 3.2. pH

Table 2 shows the pH according to the fermentation temperature of the fermented sausages added with the strains isolated from Korean fermented foods. The pH of the all groups showed a tendency to decrease as the fermentation and drying periods elapsed. The pH of the fermented sausage at the initial fermentation stage and drying was reported to decrease due to acidification, as the lactic acid bacteria grew, showing similar results [30]. However, GD20 and ND20 showed a significant increase in pH on day 24, and CS20 and ND25 on day 31 (*p* < 0.05). The microbial population analysis results in this study revealed that *Lactobacillus* spp. entered a stationary and a death phase as the fermentation and drying periods elapsed, whereas *Staphylococcus* spp., yeast, and mold exhibited an exponential phase. *Staphylococcus* spp., yeast, and mold exhibited higher pH levels than that of lactic acid bacteria. In addition, microorganisms in fermented sausages break down proteins to produce VBN such as NH_3_, and the produced material exhibited a high level of pH, increasing the pH of the fermented sausage [31]. After day 10, the pH of the 25 °C group showed a significantly lower value than that of the 20 °C group (*p* < 0.05). According to microbial population analysis, *Lactobacillus* spp. showed a higher growth rate at 25 °C than at 20 °C. Accordingly, the 25 °C group exhibited strong acidification compared to that of the 20 °C group. In addition, pH of the GD group was significantly lower than that of the ND group in all periods (*p* < 0.05). Canon et al. [32] reported that lactic acid bacteria in fermented meat products could inhibit the growth of numerous pathogens and spoilage microorganisms by lowering pH and generating various antibacterial compounds. Therefore, producing fermented sausages using GD strains isolated from Korean fermented foods is safer at 25 °C.

### 3.3. Moisture Content

An intermediate moisture food has improved storage properties by lowering moisture to a level that inhibits the growth of microorganisms, with a fermented sausage being a typical example [33]. Table 3 shows the moisture content according to the fermentation temperature of fermented sausages using strains isolated from Korean fermented foods. The moisture content of the control and all treatment groups showed a decrease as the fermentation and drying periods elapsed. Moisture loss may have occurred due to the growth of microorganisms in the fermented sausage and the fermentation and drying process for 31 days. After day 17, the 25 °C group showed a significantly lower moisture content than that of the 20 °C group (*p* < 0.05). The 25 °C group was fermented at a higher temperature than the 20 °C group, with the drying process performed while gradually decreasing the temperature. The initial fermentation and drying temperatures were high, leading to quicker moisture loss. According to the Korean Food Code, the final moisture content of dried fermented sausages is regulated at 35% [34]. The moisture contents of ND20 and GD25 were significantly higher than that of GD20 and ND25, respectively (*p* < 0.05). However, in the fermented sausages using strains isolated from Korean fermented foods, the moisture content of the final product was within 35% of both the 20 °C and 25 °C groups. Both the GD and the ND groups were determined as suitable to produce fermented sausages at temperatures of 20 °C and 25 °C.

### 3.4. Color

Table 4 shows the color according to the fermentation temperature of the fermented sausages added with the strains isolated from Korean fermented foods.

The lightness was significantly higher in the 20 °C group than that in the 25 °C group for all periods (*p* < 0.05). During the drying process, the amount of light scattered in fermented sausages decreased as the surface moisture eluted. As the 25 °C group showed a lower moisture content than that of 20 °C group, the amount of scattered light was low leading to the low lightness [35]. Redness decreased as the fermentation and drying period elapsed, but there was no significant difference between the 20 °C group and the 25 °C group in most periods (*p* > 0.05). The decrease in redness occurs as the myoglobin in the fermented sausage is exposed to oxygen and turns brown as it is converted to myoglobin and metmyoglobin [36]. The yellowness increased as the fermentation and drying periods increased, significantly higher in the 25 °C group than in the 20 °C group (*p* < 0.05). Myoglobin forms sulfmyoglobin, a green substance, after hydrogen sulfide produced during proteolysis is combined with oxygen [37]. As green pigment (180°) is generated from red (0°), which is the hue angle of meat, it shows a value close to yellow (90°), increasing the yellowness [38]. As the 25 °C group showed a higher level of microorganisms than that of the 20 °C group, a large amount of hydrogen sulfide was generated, indicating that yellowness denoted a higher value.

### 3.5. TBARS 

Table 5 shows the TBARS values according to the fermentation temperature of fermented sausages added with strains isolated from Korean fermented foods. After day 17, the TBARS values of the control and all treatment groups were significantly higher in the 25 °C group than that of the 20 °C group (*p* < 0.05), and the final TBARS values of the 20 °C and 25 °C groups were 1.68–4.35 MDA/kg and 5.23–7.58 MDA/kg, respectively. Fat is broken down into fatty acids such as saturated fatty acids (SFA), monounsaturated fatty acids (MUFA), and polyunsaturated fatty acids (PUFA) by endogenous or microbial lipases, with long-chain fatty acids such as PUFA oxidized first [39,40]. When these fats are exposed to oxygen, primary oxidation produces hydroperoxides, which are primary products. The primary products are exposed continuously to oxygen to start secondary oxidation and are converted into secondary products such as aldehydes, ketones, epoxides, hydroxy compounds, oligomers, and polymers [41]. The TBARS value is determined by the level of malondialdehyde (MDA), which accounts for most of the aldehydes, as the TBARS value is higher with more secondary products [42]. Therefore, the TBARS value was higher in the 25 °C group, which showed a high level of microorganisms, than in the 20 °C group because there were more aldehydes produced. Moreover, the ND group showed the most dramatic difference in TBARS values according to the fermentation temperature compared to the CS and GD groups on day 31. 

These results mean that the ND strain has the largest difference in the amount of aldehydes produced for reasons unknown in this study. This part will be studied while analyzing fatty acid, volatile composition data in future studies. In general, a high level of TBARS value has a negative effect on food; however, pleasant aromas may be expressed in fermented meat products [43]. Domínguez et al. [44] reported that hexanal, produced during fat oxidation of fermented meat products, plays an important role in developing flavor in the drying phase. As the TBARS value was high in sausages with strains isolated from Korean fermented foods fermented at 25 °C, the flavor was expected to be stronger.

### 3.6. VBN

In general, VBN measurement is performed to confirm the degree of food contamination, but the VBN value in fermented meat products is an indicator that can confirm the degree of protein degradation [45]. Table 6 shows the VBN values according to the fermentation temperature of fermented sausages with the isolated strains from Korean fermented foods. After day 10, the VBN values of the control and treatment groups were significantly higher in the 25 °C group than that in the 20 °C group (*p* < 0.05), and the VBN values of the ND group were significantly higher than that of the CS group in all periods. In addition, the VBN value of the final product was 6.44 to 11.57 mg%. The microbial group (LAB, *Staphylococcus* spp., yeast, and mold) formed in fermented sausages decomposes proteins to produce non-protein nitrogen compounds such as NH_3_, H_2_S, and CH_3_CH_2_S, thereby increasing the VBN value [46]. Therefore, the same results as in this study were obtained as the microorganisms of the 25 °C and ND groups degraded more proteins than that of the 20 °C and CS groups, respectively. According to the Korean Food Code, the final VBN content of a final meat product is regulated at 20 mg% or less [34]. In this study, the VBN value of the 25 °C group was higher than that of the 20 °C group, but both were within 20 mg%, indicating that fermentation at 20 °C and 25 °C was suitable for both the GD and ND groups in fermented sausage production.

### 3.7. Electronic Nose (E-Nose)

**Figure 1:**
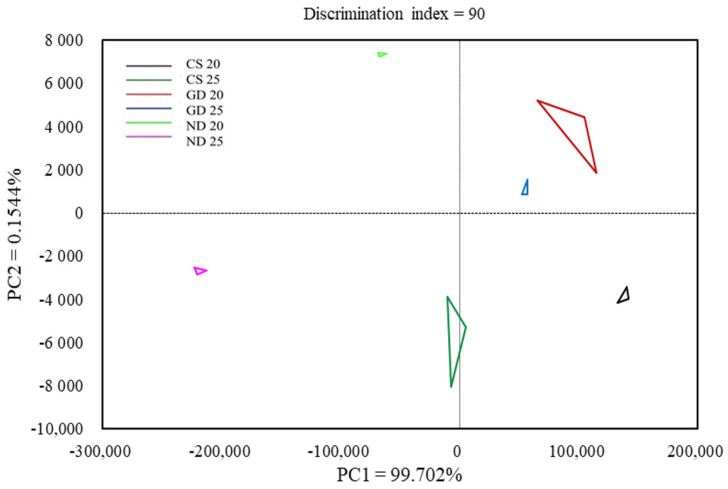
Principal component analysis of fermented sausage using strains isolated from Korean fermented food according to fermentation temperature. CS, commercial starter culture; GD, *Pediococcus pentosaceus* SMFM2016-GK1 + *Debaryomyces hansenii* SMFM2021-D1; ND, *P. pentosaceus* SMFM2016-NK3 + *D. hansenii* SMFM2021.

Figure 1 and Table 7 show the results of electronic nose testing according to the fermentation temperature of fermented sausages added with strains isolated from Korean fermented foods as PCA and volatile compounds. The GD and ND groups were located on the right and left side of the *y*-axis, respectively (Figure 1). Hu et al. [47] reported that lactic acid bacteria were mainly involved in the flavor formation of fermented sausages, and different aroma profiles are a result of the differences in the proteolysis and lipolysis levels of GD and ND. GD20 and GD25 showed a close aroma profile compared to that of ND20 and ND25, which was determined as an index to judge that GD had less flavor change according to fermentation temperature compared to ND.

In the control and treatment groups, 11 volatile compounds related to fermentation and aging were detected (Table 7). The volatile compound showing the highest value was identified as ethanol, which was the highest in CS20, followed by GD20, GD25, CS25, ND20, and ND25. Wen et al. [48] reported that ethanol was produced during carbohydrate fermentation of yeast strains. In this study, the YMC value of the 20 °C group was higher than that of the 25 °C group, which may have been affected by the ethanol level. Acetoin was the highest in GD25, followed by CS25, ND25, ND20, GD20, and CS20. Acetoin is a compound produced by carbohydrate fermentation of LAB and *Staphylococcus* spp., which produces buttery and sweet odors as it is converted to diacetyl (2,3-butanedione) upon oxidation [49,50]. In this study, the LABC and STPC values of the 25 °C group were higher than those of the 20 °C group, indicating a high acetoin content in the 25 °C group. As GD25 showed higher acetoin levels than that of ND25, inoculation of GD during production of fermented sausage is expected to produce a stronger flavor of fermented sausage than inoculation of ND.

## 4. Conclusions

This study aims at identifying the optimal fermentation temperature for dry-fermented sausage using strains isolated from Korean fermented foods. According to microbial population analysis, the aerobic bacteria *Lactobacillus* spp., and *Staphylococcus* spp., showed higher growth after fermentation at 25 °C compared to 20 °C, but yeast and mold showed lower growth. According to TBARS analysis, the 25 °C group is predicted to show a higher value than the 20 °C group, with a strong flavor. Following electronic nose testing, a large amount of “acetoin” was detected in GD25 compared to that of ND25, which can enhance the flavor of fermented sausage. Therefore, inoculating GD and fermenting at 25 °C is appropriate when manufacturing fermented sausage inoculated with a strain isolated from Korean fermented foods.

## Figures and Tables

**Table 1:** Microbial population (express as log CFU/g) of fermented sausage using strains isolated from Korean fermented foods according to fermentation temperature.

Trait	Time (Days)	CS ^5^		GD ^6^		ND ^7^		SEM ^8^
		20 °C	25 °C	20 °C	25 °C	20 °C	25 °C	
AC ^1^	0	4.33 ^Cb^	4.33 ^Cb^	4.86 ^Ca^	4.86 ^Ca^	5.02 ^Ca^	5.02 ^Da^	0.10
	3	7.91 ^Bc^	9.07 ^Aa^	7.70 ^Bd^	7.84 ^Ac^	7.91 ^Ac^	8.31 ^Ab^	0.10
	10	7.97 ^Bc^	8.80 ^Aa^	8.07 ^Ac^	7.96 ^Ac^	7.97 ^Ac^	8.42 ^Ab^	0.21
	17	8.38 ^Aa^	8.29 ^Ba^	7.84 ^ABb^	7.77 ^Ab^	7.78 ^ABb^	8.14 ^Ba^	0.19
	24	8.10 ^ABa^	8.11 ^Ba^	7.71 ^Bb^	7.49 ^Bb^	7.67 ^Bb^	8.04 ^Ba^	0.16
	31	7.89 ^Ba^	8.10 ^Ba^	7.56 ^Bb^	7.49 ^Bb^	7.58 ^Bb^	7.86 ^Ba^	0.16
	SEM	0.20	0.11	0.19	0.15	0.09	0.17	
LABC ^2^	0	3.96 ^Db^	3.96 ^Bb^	4.70 ^Ca^	4.70 ^Da^	4.56 ^Ea^	4.56 ^Da^	0.15
	3	8.47 ^ABb^	9.21 ^Aa^	7.57 ^Bc^	8.89 ^Aa^	6.83 ^Dd^	8.34 ^Cb^	0.23
	10	8.66 ^Abc^	9.13 ^Aa^	8.00 ^Ad^	8.86 ^ABb^	8.48 ^Ac^	8.78 ^Ab^	0.15
	17	8.67 ^Ab^	9.15 ^Aa^	7.96 ^Ac^	8.74 ^ABCb^	7.98 ^ABc^	8.70 ^ABb^	0.15
	24	8.19 ^Bc^	9.18 ^Aa^	7.97 ^Ad^	8.71 ^BCb^	7.54 ^BCe^	8.66 ^ABb^	0.13
	31	7.85 ^Cc^	9.14 ^Aa^	7.52 ^Bd^	8.69 ^Cb^	7.45 ^Cd^	8.53 ^Bb^	0.16
	SEM	0.16	0.26	0.22	0.10	0.10	0.13	
STPC ^3^	0	3.44 ^Ca^	3.44 ^Ca^	3.60 ^Da^	3.60 ^Da^	3.61 ^Ca^	3.61 ^Ca^	0.09
	3	3.59 ^Cd^	3.70 ^Cd^	5.36 ^Cc^	6.02 ^Cb^	5.55 ^Bc^	6.43 ^Ba^	0.19
	10	3.71 ^BCd^	4.47 ^Bc^	5.94 ^Bb^	6.21 ^Cb^	5.88 ^ABb^	7.24 ^Aa^	0.33
	17	3.84 ^BCe^	4.47 ^Bd^	6.03 ^Bc^	6.82 ^Bb^	6.10 ^Ac^	7.24 ^Aa^	0.19
	24	4.25 ^Bc^	4.49 ^Bc^	6.09 ^Bb^	7.05 ^ABa^	6.28 ^Ab^	7.25 ^Aa^	0.27
	31	4.94 ^Ac^	4.95 ^Ac^	6.42 ^Ab^	7.21 ^Aa^	6.37 ^Ab^	7.51 ^Aa^	0.23
	SEM	0.16	0.30	0.26	0.18	0.18	0.23	
YMC ^4^	0	4.76 ^Fb^	4.76 ^Cb^	5.27 ^Da^	5.27 ^Da^	5.09 ^Ca^	5.09 ^Da^	0.11
	3	5.21 ^Ed^	5.64 ^Bc^	5.69 ^CDc^	6.13 ^Cb^	6.57 ^Ba^	6.08 ^Cb^	0.11
	10	5.91 ^Db^	6.05 ^ABb^	6.15 ^Cb^	6.33 ^Cab^	6.63 ^Ba^	6.17 ^Cb^	0.21
	17	6.42 ^Cab^	6.06 ^ABb^	7.21 ^Ba^	6.36 ^Cab^	6.72 ^Bab^	6.61 ^Bab^	0.33
	24	6.74 ^Bb^	6.03 ^ABc^	7.47 ^Ba^	6.91 ^Bb^	6.85 ^Bb^	6.63 ^Bb^	0.24
	31	7.17 ^Ab^	6.51 ^Ac^	8.05 ^Aa^	7.52 ^Aab^	7.52 ^Aab^	7.58 ^Aab^	0.27
	SEM	0.31	0.25	0.10	0.20	0.10	0.32	

^1^ AC, aerobic bacteria plate count. ^2^ LABC, *Lactobacillus* spp. plate count. ^3^ STPC, *Staphylococcus* spp. plate count. ^4^ YMC, yeast and mold plate count. ^5^ CS, commercial starter culture. ^6^ GD, *Pediococcus pentosaceus* SMFM2016-GK1 + *Debaryomyces hansenii* SMFM2021-D1. ^7^ ND, *P. pentosaceus* SMFM2016-NK3 + *D. hansenii* SMFM2021. ^8^ SEM, standard error of mean (*n* = 6). ^A–F^ The means in the same column with different letters are significantly different (*p* < 0.05). ^a–e^ The means in the same row with different letters are significantly different (*p* < 0.05).

**Table 2:** pH of fermented sausage using strains isolated from Korean fermented foods according to fermentation temperature.

Time (Days)	CS ^1^		GD ^2^		ND ^3^		SEM ^4^
	20 °C	25 °C	20 °C	25 °C	20 °C	25 °C	
0	5.93 ^Aa^	5.93 ^Aa^	5.96 ^Aa^	5.96 ^Aa^	5.94 ^Aa^	5.94 ^Aa^	0.02
3	5.67 ^Bc^	4.89 ^Be^	5.83 ^Ba^	5.29 ^Bd^	5.78 ^Cab^	5.73 ^Bbc^	0.06
10	4.79 ^De^	4.64 ^Cf^	5.38 ^Eb^	5.03 ^Cd^	5.78 ^Ca^	5.12 ^Dc^	0.04
17	4.75 ^De^	4.66 ^Cf^	5.33 ^Eb^	4.94 ^Cd^	5.70 ^Da^	5.09 ^Dc^	0.05
24	4.79 ^De^	4.69 ^Cf^	5.61 ^Db^	5.07 ^Cd^	5.82 ^Ba^	5.19 ^Dc^	0.06
31	4.99 ^Ce^	4.67 ^Cf^	5.76 ^Cb^	5.03 ^Cd^	5.96 ^Aa^	5.53 ^Cc^	0.05
SEM	0.05	0.05	0.05	0.07	0.02	0.05	

^1^ CS, commercial starter culture. ^2^ GD, *Pediococcus pentosaceus* SMFM2016-GK1 + *Debaryomyces hansenii* SMFM2021-D1. ^3^ ND, *P. pentosaceus* SMFM2016-NK3 + *D. hansenii* SMFM2021. ^4^ SEM, standard error of mean (*n* = 6). ^A–E^ The means in the same column with different letters are significantly different (*p* < 0.05). ^a–f^ The means in the same row with different letters are significantly different (*p* < 0.05).

**Table 3:** Moisture content of fermented sausage using strains isolated from Korean fermented foods according to fermentation temperature.

Time (Days)	CS ^1^		GD ^2^		ND ^3^		SEM ^4^
	20 °C	25 °C	20 °C	25 °C	20 °C	25 °C	
0	64.71 ^Aa^	64.71 ^Aa^	64.71 ^Aa^	64.71 ^Aa^	64.71 ^Aa^	64.71 ^Aa^	2.06
3	57.75 ^Ba^	54.87 ^Bbc^	57.67 ^Ba^	53.01 ^Bc^	56.97 ^Bab^	54.45 ^Bc^	1.41
10	48.24 ^Ca^	41.23 ^Cc^	43.74 ^Cb^	39.70 ^Cd^	44.74 ^Cb^	43.97 ^Cb^	0.84
17	42.29 ^Da^	36.53 ^Db^	42.09 ^Ca^	36.47 ^Db^	41.43 ^Da^	35.33 ^Db^	1.04
24	37.84 ^Ea^	25.81 ^Ec^	38.14 ^Da^	31.35 ^Eb^	39.62 ^Da^	23.58 ^Ec^	1.42
31	35.82 ^Ea^	23.39 ^Ed^	32.49 ^Eb^	28.86 ^Fc^	34.53 ^Ea^	23.23 ^Ed^	1.14
SEM	1.16	1.63	1.36	1.09	1.34	1.33	

^1^ CS, commercial starter culture. ^2^ GD, *Pediococcus pentosaceus* SMFM2016-GK1 + *Debaryomyces hansenii* SMFM2021-D1. ^3^ ND, *P. pentosaceus* SMFM2016-NK3 + *D. hansenii* SMFM2021. ^4^ SEM, standard error of mean (*n* = 6). ^A–F^ The means in the same column with different letters are significantly different (*p* < 0.05). ^a–d^ The means in the same row with different letters are significantly different (*p* < 0.05).

**Table 4:** Color of fermented sausage using strains isolated from Korean fermented foods according to fermentation temperature.

Trait	Time (Days)	CS ^1^		GD ^2^		ND ^3^		SEM ^4^
		20 °C	25 °C	20 °C	25 °C	20 °C	25 °C	
CIE L^*^	0	61.10 ^Aa^	61.10 ^Aa^	61.10 ^Aa^	61.10 ^Aa^	61.10 ^Aa^	61.10 ^Aa^	0.36
	3	58.20 ^Ba^	52.92 ^Bd^	56.04 ^Bab^	52.27 ^Bd^	54.85 ^Bbc^	53.30 ^Bcd^	1.30
	10	58.57 ^Ba^	53.82 ^Bb^	55.37 ^Bb^	50.38 ^BCc^	54.47 ^Bb^	48.27 ^Cc^	1.44
	17	56.33 ^Ca^	47.82 ^Cb^	54.92 ^Ba^	48.76 ^Cb^	55.35 ^Ba^	44.7 ^Dc^	1.67
	24	55.13 ^Ca^	47.38 ^Cb^	54.32 ^Ba^	48.25 ^Cb^	53.85 ^Ba^	45.33 ^Dc^	1.55
	31	51.73 ^Da^	47.65 ^Cb^	51.70 ^Ca^	45.82 ^Dbc^	50.70 ^Ca^	45.28 ^Dc^	1.50
	SEM	1.20	1.75	1.12	1.18	1.60	0.96	
CIE a^*^	0	7.00 ^Aa^	7.00 ^Aa^	7.00 ^Aa^	7.00 ^Aa^	7.00 ^Aa^	7.00 ^Aa^	0.20
	3	6.97 ^Aa^	4.60 ^Bc^	6.30 ^ABab^	6.27 ^ABab^	6.13 ^ABab^	5.60 ^Bbc^	0.62
	10	5.75 ^Aa^	4.78 ^Ba^	5.80 ^BCa^	5.82 ^Ba^	5.50 ^BCa^	4.88 ^Ca^	0.83
	17	6.37 ^Aa^	4.60 ^Bb^	4.97 ^CDb^	4.28 ^Cb^	5.08 ^Cb^	4.70 ^Cb^	0.62
	24	6.28 ^Aa^	3.72 ^Bcd^	4.58 ^CDbc^	3.67 ^Cd^	4.60 ^CDb^	4.58 ^Cbc^	0.60
	31	4.00 ^Ba^	3.58 ^Ba^	4.23 ^Da^	3.80 ^Ca^	3.70 ^Da^	4.25 ^Ca^	0.95
	SEM	0.73	0.78	0.64	0.72	0.61	0.34	
CIE b^*^	0	6.97 ^Ea^	6.97 ^Ea^	6.97 ^Ba^	6.97 ^Ca^	6.97 ^Ca^	6.97 ^Ba^	0.15
	3	8.00 ^Db^	9.45 ^Da^	8.30 ^Ab^	9.35 ^Ba^	8.38 ^Bb^	9.47 ^Aa^	0.39
	10	8.14 ^CDb^	9.72 ^CDa^	8.53 ^Ab^	9.40 ^Ba^	8.40 ^ABb^	9.80 ^Aa^	0.52
	17	8.46 ^BCc^	10.40 ^BCa^	8.43 ^Ac^	9.40 ^Bb^	8.48 ^ABc^	9.95 ^Aa^	0.32
	24	8.85 ^ABc^	10.33 ^ABa^	8.43 ^Ac^	9.47 ^Bb^	8.53 ^ABc^	9.93 ^Aa^	0.36
	31	9.25 ^Ac^	11.33 ^Aa^	8.68 ^Ad^	10.08 ^Ab^	8.73 ^Ad^	10.00 ^Ab^	0.31
	SEM	0.34	0.42	0.38	0.15	0.31	0.26	

^1^ CS, commercial starter culture. ^2^ GD, *Pediococcus pentosaceus* SMFM2016-GK1 + *Debaryomyces hansenii* SMFM2021-D1. ^3^ ND, *P. pentosaceus* SMFM2016-NK3 + *D. hansenii* SMFM2021. ^4^ SEM, standard error of mean (*n* = 6). ^A–E^ The means in the same column with different letters are significantly different (*p* < 0.05). ^a–d^ The means in the same row with different letters are significantly different (*p* < 0.05).

**Table 5:** Thiobarbituric acid reactive substance (malondialdehyde/kg sample) of fermented sausage using strains isolated from Korean fermented foods according to fermentation temperature.

Time (Days)	CS ^1^		GD ^2^		ND ^3^		SEM ^4^
	20 °C	25 °C	20 °C	25 °C	20 °C	25 °C	
0	0.61 ^Da^	0.61 ^Ea^	0.61 ^Da^	0.61 ^Da^	0.61 ^Ca^	0.61 ^Ea^	0.04
3	0.58 ^Dc^	1.44 ^Da^	0.66 ^Dbc^	1.41 ^Da^	0.67 ^Cbc^	0.97 ^Db^	0.12
10	0.87 ^Dc^	4.90 ^Ca^	0.72 ^Dc^	3.37 ^Db^	0.76 ^Cc^	1.09 ^Dc^	0.23
17	2.70 ^Cd^	5.06 ^Ca^	1.47 ^Ce^	4.49 ^Cb^	1.13 ^Be^	3.27 ^Cc^	0.22
24	3.21 ^Bd^	7.06 ^Ba^	1.63 ^Be^	4.64 ^Bb^	1.30 ^Be^	3.98 ^Bc^	0.23
31	4.35 ^Ad^	7.58 ^Aa^	2.12 ^Ae^	5.23 ^Ac^	1.68 ^Af^	6.71 ^Ab^	0.18
SEM	0.18	0.11	0.07	0.37	0.08	0.21	

^1^ CS, commercial starter culture. ^2^ GD, *Pediococcus pentosaceus* SMFM2016-GK1 + *Debaryomyces hansenii* SMFM2021-D1. ^3^ ND, *P. pentosaceus* SMFM2016-NK3 + *D. hansenii* SMFM2021. ^4^ SEM, standard error of mean (*n* = 6). ^A–E^ The means in the same column with different letters are significantly different (*p* < 0.05). ^a–f^ The means in the same row with different letters are significantly different (*p* < 0.05).

**Table 6:** Volatile basic nitrogen (mg%) of fermented sausage using strains isolated from Korean fermented foods according to fermentation temperature.

Time (Days)	CS ^1^		GD ^2^		ND ^3^		SEM ^4^
	20 °C	25 °C	20 °C	25 °C	20 °C	25 °C	
0	2.18 ^Da^	2.18 ^Fa^	2.18 ^Da^	2.18 ^Ea^	2.18 ^Da^	2.18 ^Fa^	0.21
3	4.20 ^Cc^	4.82 ^Ec^	4.63 ^Cc^	6.33 ^Da^	5.67 ^Cb^	6.72 ^Ea^	0.29
10	5.60 ^Bd^	6.65 ^Dc^	7.50 ^Bb^	7.95 ^Cab^	7.06 ^Bc^	8.36 ^Da^	0.35
17	5.94 ^ABe^	7.22 ^Cd^	7.84 ^ABc^	8.66 ^Bb^	7.09 ^Bd^	9.18 ^Ca^	0.18
24	6.16 ^Ae^	7.99 ^Bc^	7.84 ^ABcd^	9.13 ^ABb^	7.39 ^Bd^	10.42 ^Ba^	0.31
31	6.44 ^Ad^	9.24 ^Ab^	8.36 ^Ac^	9.56 ^Ab^	8.46 ^Ac^	11.57 ^Aa^	0.40
SEM	0.30	0.23	0.30	0.36	0.24	0.32	

^1^ CS, commercial starter culture. ^2^ GD, *Pediococcus pentosaceus* SMFM2016-GK1 + *Debaryomyces hansenii* SMFM2021-D1. ^3^ ND, *P. pentosaceus* SMFM2016-NK3 + *D. hansenii* SMFM2021. ^4^ SEM, standard error of mean (*n* = 6). ^A–F^ The means in the same column with different letters are significantly different (*p* < 0.05). ^a–e^ The means in the same row with different letters are significantly different (*p* < 0.05).

**Table 7:** Volatile compounds of fermented sausage using strains isolated from Korean fermented food according to fermentation temperature.

Expected Volatile Compounds	CS ^1^		GD ^2^		ND ^3^		SEM ^4^
	20 °C	25 °C	20 °C	25 °C	20 °C	25 °C	
Acetaldehyde	4774.68	860.51	4193.42	5824.64	1693.92	2312.24	24.31
Ethanol	258,474.64	166,689.25	230,930.89	205,370.92	126,849.58	27,718.30	820.27
Propan-2-one	16,124.12	13,101.51	9816.87	9347.94	5343.18	10,098.84	88.85
Butanal	282.15	1959.60	499.20	2293.30	824.75	691.50	13.31
Butan-2-one	2223.46	1205.25	1084.56	1172.71	1237.28	828.16	20.77
Ethyl acetate	6869.33	5603.10	3333.27	4883.84	2849.78	488.18	22.17
Acetoin	139.09	3028.29	440.63	4098.40	1052.01	885.65	24.51
Hexanal	3045.24	2207.68	1033.54	869.48	358.95	1005.81	14.67
Hexanoic acid	709.14	1856.97	594.75	1766.51	535.34	1687.16	7.19
p-Cymene	597.16	1848.63	508.64	1899.63	453.26	1822.97	6,28
Limonene	697.44	1725.32	576.28	1683.38	507.83	1717.92	5.03

^1^ CS, commercial starter culture. ^2^ GD, *Pediococcus pentosaceus* SMFM2016-GK1 + *Debaryomyces hansenii* SMFM2021-D1. ^3^ ND, *P. pentosaceus* SMFM2016-NK3 + *D. hansenii* SMFM2021. ^4^ SEM, standard error of mean (*n* = 6).

## Data Availability

Data is contained within the article.
